# A review of the treatment options for skin rash induced by EGFR-targeted therapies: Evidence from randomized clinical trials and a meta-analysis

**DOI:** 10.2478/raon-2013-0014

**Published:** 2013-05-21

**Authors:** Janja Ocvirk, Steffen Heeger, Philip McCloud, Ralf-Dieter Hofheinz

**Affiliations:** 1 Institute of Oncology, Ljubljana, Slovenia; 2 Merck KGaA, Darmstadt, Germany; 3 McCloud Consulting Group, Sydney, Australia; 4 Universitätsmedizin Mannheim, University of Heidelberg, Germany

**Keywords:** acne-like skin rash, cetuximab, antibiotics, erlotinib, gefitinib, panitumumab, vitamin K

## Abstract

**Background:**

Agents targeting the epidermal growth factor receptor (EGFR) are amongst the most extensively used of the targeted agents in the therapy of some of the most common solid tumors. Although they avoid many of the classic side effects associated with cytotoxic chemotherapy, they are associated with unpleasant cutaneous toxicities which can affect treatment compliance and impinge on patient quality of life. To date, despite a plethora of consensus recommendations, expert opinions and reviews, there is a paucity of evidence-based guidance for the management of the skin rash that occurs in the treatment of patients receiving EGFR-targeted therapies.

**Methods:**

A literature search was conducted as a first step towards investigating not only an evidence-based approach to the management of skin rash, but also with a view to designing future randomized trials.

**Results:**

The literature search identified seven randomized trials and a meta-analysis was conducted using the data from four of these trials involving oral antibiotics. The meta-analysis of the data from these four trials suggests that prophylactic antibiotics might reduce the relative risk of severe rash associated with EGFR-targeted agents by 42–77%. Vitamin K cream was also identified as having a potential role in the management EGFR-targeted agent induced rash.

**Conclusions:**

This review and meta-analysis clearly identify the need for further randomized studies of the role of oral antibiotics in this setting. The results of the ongoing randomized trials of the topical application of vitamin K cream plus or minus doxycycline and employing prophylactic versus reactive strategies are eagerly awaited.

## Introduction

Recognition of the importance of the epidermal growth factor receptor (EGFR [HER1]) in tumorigenesis and tumor progression^1^–^5^ led to the development of EGFR-targeted therapies, including the monoclonal antibodies (mAbs) cetuximab and panitumumab, and the EGFR tyrosine kinase inhibitors (TKIs) gefitinib, erlotinib and lapatinib, for use in the therapy of a range of solid tumors including those of the colon and rectum, head and neck, lung, pancreas and breast.^6^Although, the toxicity profiles of EGFR-targeted therapies, across the different malignancies, largely exclude many of the severe side-effects observed with cytotoxic agents (*e.g*. hematological side-effects and hair loss), they are associated with the development of cutaneous toxicities.^7^–^12^ The earliest and most common of these, for both classes of EGFR-targeted agents, is a papulopustular skin rash^13^, although the variety of terms used to describe skin rash has made direct comparisons of its incidence between trials and agents complicated.^10^ Other well-documented cutaneous toxicities associated with these agents include xerosis, pruritus, and specific hair and nail changes (Table 1).^8^,^12^,^14^ An overview of the incidence of skin toxicities with different EGFR-targeted agents, according to their licensed indications is presented in Table 2.

Generally, the cutaneous toxicities associated with these targeted agents are classified as mild to moderate, but if left untreated they can potentially affect both patient quality of life (QoL)^15^–^17^ and treatment compliance.^18^,^19^ They can also predispose the skin to bacterial, fungal, or viral infections. Also, given the association between the manifestation of cutaneous toxicities and the clinical efficacy of EGFR-targeted treatment approaches,^10^,^20^–^24^ it is becoming increasingly clear that therapeutic and preventive strategies need to be, and should be, adopted in the management of such toxicities to facilitate treatment continuation whilst maintaining maximal patient tolerability and the avoidance of treatment delays and interruptions.

This narrative review will focus on the reactive and prophylactic approaches, for the improved management of the cutaneous toxicities, specifically rash, induced by EGFR-targeted agents, investigated in recent randomized clinical trials. As a meta-analysis is a systematic approach to identification and abstraction of critical information from different randomized^25^, controlled trials, we also made the meta-analysis of the data obtained from four of these recent randomized trials investigating the use of antibiotics.

## Methods

A search of Medline and of the abstracts on oncology meeting databases provided the literature sources on which this review is based. This search identified four manuscripts, reporting the data from four individual randomized trials on the skin toxicities caused by EGFR targeted agents that could be combined in a meta-analysis of skin toxicity. The primary results from the meta-analysis of these four trials will also be reported.

## Results

### EGFR-targeted agent induced skin toxicity

#### Grading and treatment strategies

To date, the National Cancer Institute Common Terminology Criteria for Adverse Events (NCI-CTCAE) version 3.0 grading scale, published in 2006, is the scale that has been used most often for grading the skin toxicities induced by EGFR-targeted therapies. However, this scale is not optimal for grading the skin toxicities, and in particular the papulopustular acneiform rash, induced by this class of agent. As a consequence an updated version of this grading scale^26^, and other grading systems have been proposed.^27^,^28^ In addition, the lack of a standardized approach to both the grading and management of skin reactions induced by EGFR-targeted therapies has been identified.^29^

It is against this background that at least five sets of consensus recommendations have been published^8^,^9^,^29^–^31^, together with expert opinions and recommendations for the treatment of the skin toxicities associated with EGFR-targeted therapies in general^12^,^19^,^26^,^33^,^34^, and the radiation dermatitis seen in patients with locally advanced squamous cell carcinoma of the head and neck (SCCHN) receiving cetuximab concomitantly with radiotherapy^35^, in particular. There have also been several reviews.^10^,^11^,^36^–^38^ Most have detailed strategies utilizing the topical application of emollient and antibiotic and steroid creams, administration of systemic steroids and antibiotics, avoidance of sun exposure, and the use of high-protection factor sun creams, for the management of skin rash. To date, there has been at least one randomized trial of the prophylactic use of sunscreen^39^,^40^, five randomized trials of oral antibiotics (Table 3)^16^,^41^–^44^, and one of the prophylactic use of topical pimecrolimus, an immunomodulator^45^, developed for the treatment of inflammatory skin disease. These randomized studies were conducted with a view to providing a much needed evidence base for the refinement of the treatment approaches used in the management of the cutaneous toxicities experienced by patients receiving EGFR-targeted therapies. They are discussed in detail below according to individual category.

### Randomized trials

#### Antibiotics and skin rash

Standard tetracycline is commonly used for the treatment of acne, and the similarity between acne and EGFR-targeted agent-induced skin rash suggested a possible role for tetracycline in the treatment or prevention of the skin rash that occurs following treatment with these agents.^41^ It was also thought that the anti-inflammatory effects of tetracycline might provide effective rash palliation. Thus, following on from several case/small studies^46^–^48^, there have been five randomized trials of the use of standard tetracycline and tetracycline-class (minocycline and doxycycline) antibiotics in the treatment of skin rash in patients receiving EGFR-targeted therapies.

#### Tetracycline

The first of these was a randomized, placebo-controlled, phase III trial (N03CB), conducted in the US by the North Central Cancer Treatment Group (NCCTG). In this trial, 61 cancer patients (31 lung, 16 CRC and 15 other malignancies), starting treatment with an EGFR-targeted agent (cetuximab or gefitinib), were randomized to receive either tetracycline (500 mg/bid for 28 days) or placebo.^41^ The primary objective of the study was to compare the incidence of rash in patients treated with prophylactic tetracycline versus placebo. Patients were evaluated at the end of weeks 4 and 8, for performance status, adverse events and rash according to NCI-CTCAE version 3.0. In addition, the skin-specific, health-related QoL questionnaire Skindex-16 49 was used to measure the effects on patient QoL.

The incidence of rash was found to be comparable across the two arms. Sixteen (70%) patients treated with tetracycline and 22 (76%) patients treated with placebo developed physician-reported rash (p = 0.61), during the first 4 weeks. During the subsequent 4 weeks, when patients were no longer receiving either tetracycline or placebo, physician-reported rash was recorded for 13 tetracycline-treated patients (87%) and 16 placebo-treated patients (84%) (p = 0.84). There were however, some indications that tetracycline might have had an effect on rash severity. By week 4, physician-reported grade 2 rash was recorded for 4 (17%) tetracycline-treated patients compared with 16 (55%) placebo-treated patients (p = 0.04) (Table 3). By week 8, physician-reported rash was reported in 27% and 47% of tetracycline- and placebo-treated patients (p = 0.5), respectively. Results for patient-reported rash also suggested slightly better outcomes for those patients receiving tetracycline. In addition, patients treated with tetracycline also reported less itching, burning, stinging and skin irritation than patients treated with placebo.

The results of this study suggested therefore that the prophylactic use of oral tetracycline might be beneficial^41^, despite the fact that the primary endpoint of the trial was not achieved. As a consequence the authors concluded that because of the preliminary benefits observed and the general acceptance of tetracycline for the treatment of skin rashes, there should be no objection to its prescription to reduce the severity of the rash associated with EGFR-targeted agents. Unfortunately, however, the confirmatory NCCTG supplementary randomized N03CB trial conducted in 65 patients (33 in the tetracycline arm and 32 in the placebo arm) of whom >50% had metastatic colorectal cancer (mCRC) and >60% received cetuximab, failed to demonstrate any benefit conferred by tetracycline in terms of either the incidence or severity of rash in patients receiving therapy with EGFR-targeted agents.^42^

#### Tetracycline-class antibiotics

##### Minocycline

However, an earlier randomized trial of the broad-spectrum tetracycline antibiotic minocycline, in 48 patients with mCRC, showed that patients receiving prophylactic oral minocycline, on the same day as initiation of therapy with the EGFR-targeted mAb cetuximab, had a lower mean facial lesion count than those receiving placebo (Table 3).^43^

In this trial, the time course of the development of cetuximab-induced rash was comparable with that reported for previous studies,^12^,^13^ with rash developing rapidly following the start of therapy, peaking weeks 2–4, and becoming less severe as treatment continued. Clinical assessments included questionnaires and skin examinations at the end of weeks 1, 2, 4 and 8. In addition, photographic images of the face were reviewed by two independent dermatologists who were blind to the treatment arms. Total facial lesion counts were significantly lower in patients receiving minocycline weeks 1–4. Also, a lower proportion of patients in the minocycline arm reported moderate–severe itch than in the placebo arm (20% versus 50%, p = 0.05). A review of the severity of facial rash showed 4 patients (20%) with severe rash in the minocycline arm compared with 8 patients (42%) in the placebo arm (p = 0.13). The difference in the number of facial lesions and subjectively assessed itch had reduced by week 8. Prophylactic minocycline was therefore considered to be effective at reducing the severity of rash occurring during the first 4 weeks of treatment with cetuximab, but could not be recommended for use beyond 8 weeks.

##### Doxycycline

The randomized phase II trial ‘**S**kin **T**oxicity **E**valuation **P**rotocol with **P**anitumumab’ (the STEPP study) was the first to investigate the preemptive (prophylactic) versus reactive use of skin treatment strategies which included the use of moisturizers, sunscreen, topical steroids and oral doxycyline (100 mg/bid), a semi-synthetic tetracycline. Patients with mCRC (n = 95) being treated with the EGFR-targeted agent panitumumab and randomly assigned 1:1 to one or other treatment strategy, showed grade ≥2 skin toxicity to be reduced from 62% in the reactive patient group to 29% in the prophylactic patient group during the 6-week skin treatment period (odds ratio 0.3, 95% confidence interval [CI] 0.1–0.6).^16^ In addition, grade 2 and 3 skin toxicities of interest were reported in 23% and 40% of patients, and 6% and 21% of patients, for the prophylactic and reactive treatment arms, respectively. Furthermore, patients in the prophylactic treatment group reported improved QoL, between weeks 2 and 3 in particular, which was the median time to development of the first grade ≥2 skin toxicity in the reactive treatment group.^50^ The results of this study clearly support the prophylactic use of antibiotics in the management of EGFR-targeted agent-induced skin toxicity.

In another large randomized trial (CYTAR) investigating the prophylactic use of doxycycline versus placebo in 147 NSCLC patients being treated with the TKI erlotinib, those patients receiving doxycycline showed a marked reduction in the severity of Grade ≥2 erlotinib-induced folliculitis from 82% to 39% (Table 3).^44^ Patients were randomly assigned to erlotinib with or without doxycycline (100 mg/day). Serial photographs were taken for blind review. The primary objective of the study was to assess the efficacy of doxycycline in reducing the incidence of erlotinib-induced folliculitis during the first 4 months of treatment and the secondary objective to assess the impact of doxycycline on rash severity. The incidence of folliculitis was 71% in patients pretreated with doxycycline and 82% in those who were not pretreated (p = 0.117). This difference approached significance (p = 0.055) when those patients who did not actually take doxycycline were excluded from the analysis of the doxycyline arm. Doxycyline was shown to significantly reduce the severity of all erlotinib-induced folliculitis (p =<0.001), and the severity of other treatment-induced cutaneous AEs. The trial results also indicated that QoL was significantly less impaired in the doxycycline arm compared with the placebo arm at day 14 (p = 0.04).

Thus, overall, a significant reduction in the severity of skin rash was noticed in three^16^,^41^,^44^ out of the four randomized studies^16^,^41^,^2^,^44^ using NCI-CTCAE version 3.0 criteria for the evaluation. The greatest effect of the use of oral antibiotics probably occurs during the first 6–8 weeks of treatment, and the best data at this time would appear to derive from the two studies using doxycycline.^16^,^44^ Certainly, the CYTAR trial is the largest randomized trial to date to investigate the prophylactic use of a drug against EGFR-targeted agent-induced skin rash, and while the overall incidence of skin rash was not reduced, patients receiving doxycycline showed a significant reduction in the severity of their erlotinib-induced folliculitis and significantly less impairment of their QoL. Furthermore, the use of doxycycline did not affect the clinical efficacy outcomes of the EGFR-targeted therapies, namely panitumumab or erlotinib, in either trial. The data, therefore, would appear to support the prophylactic use of doxycycline for patients receiving EGFR-targeted therapy. Significantly also, with the exception of one standard tetracycline trial^42^, all the other antibiotic trials (Table 3) indicate that the skin-related QoL is better in the group receiving prophylactic antibiotics regardless of the assessment tool used. These observations are in part consistent with the data from a small meta-analysis which was conducted on four of the trials as outlined below.

#### Meta-analysis of antibiotics in the treatment of skin rash

From a review of the literature it was determined that the data from four of the above studies investigating the use of antibiotics could be combined^41^–^44^ (Table 3) in meta-analyses of rash incidence and rash severity. The randomized STEPP study^16^, with panitumumab, was excluded from the analysis because it had no placebo arm.

**FIGURE 1:**
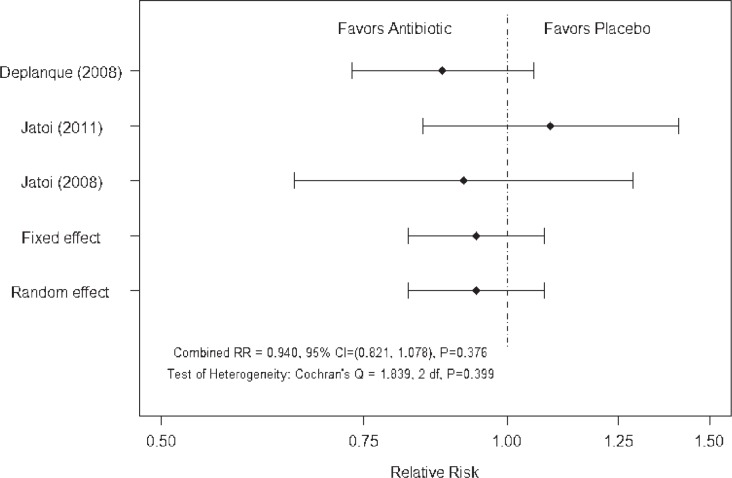
Meta-analysis of rash incidence excluding study of Scope *et al*.^43^

**FIGURE 2:**
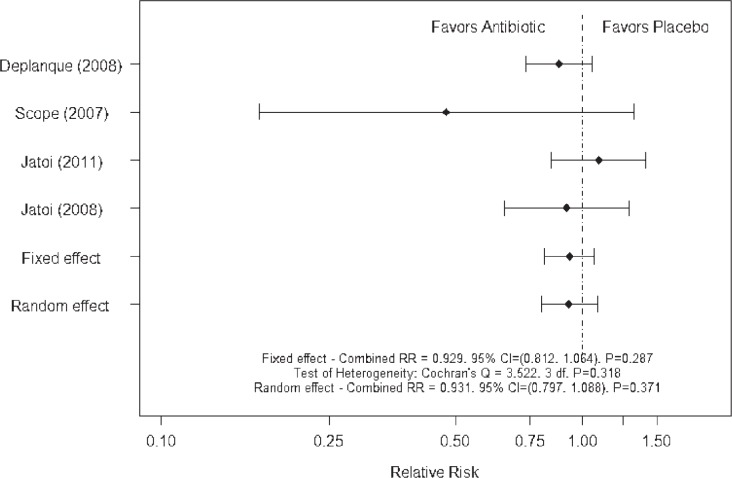
Meta-analysis of rash incidence including study of Scope *et al*.^43^

For each study the relative risk was used to measure and test the differences in rash incidence between treatments^51^, with Cochran’s Q statistic used to test the heterogeneity of the relative risks between the studies. Fixed and random effects estimates were calculated and compared.^52^ Two meta-analyses of the incidence of EGFR-targeted agent associated rash were performed, one including and one excluding the study of minocycline in patients with mCRC treated with cetuximab.^43^ This was because the relative risk for this study^43^ was based on the presence of rash rather than the incidence of moderate/severe rash. Both analyses of rash incidence, *i.e*.: without and with the minocyline study (Figures 1 and 2), showed non-significant heterogeneity between the relative risks according to Cochran’s Q statistic. The fixed and random effect analyses were identical/nearly identical and the combined relative risks were not significantly different from 1.0. Neither meta-analysis provided strong evidence that the use of an antibiotic reduced the incidence of rash associated with treatment with EGFR-targeted agents.

**FIGURE 3:**
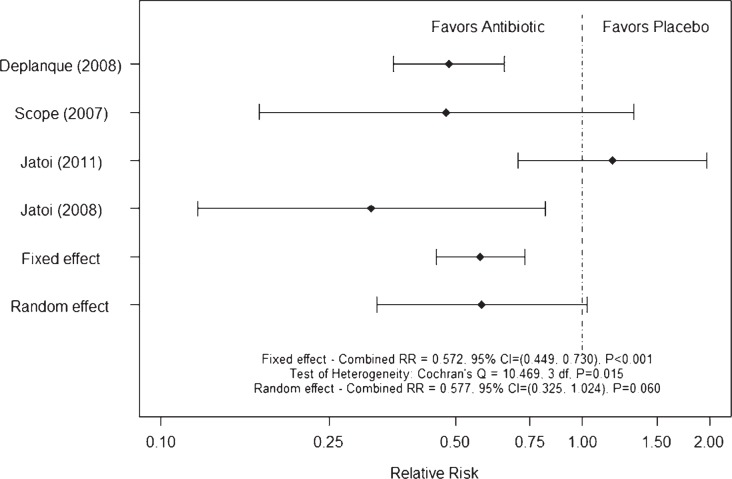
Meta-analysis of rash severity

Conversely, the meta-analysis of severe rash (Figure 3) showed significant heterogeneity between relative risks according to Cochran’s Q statistic, and the fixed and random effect analyses were not similar. The estimated relative risk that appears to have caused the significant heterogeneity is the negative tetracycline study.^42^ The estimated relative risks indicate that the risk of severe rash was reduced by 42–47% with the use of an antibiotic to control rash associated with the use of EGFR targeted agents (Figure 3). However, based on these data it is possible that tetracycline is not effective and that additional confirmatory studies are needed to validate the potential roles of doxycycline and minocycline. Thus, it is probably too early to claim doxycycline to be a ‘pseudo-standard’ in the management of patients treated with EGFR-targeted therapies.

#### Topical treatment approaches

Topical treatments that might provide an alternative treatment approach to oral antibiotics also need to be considered, as topical treatment approaches are used extensively in this setting.

#### Sunscreen and prevention of skin rash

Some patients receiving EGFR-targeted agents have been reported to show widespread erythema, infiltration and pustules in sun-exposed areas.^19^,^53^,^54^ One randomized study, N05C4, conducted in the US by NCCTG, has investigated the prophylactic use of SPF60 sunscreen in cancer patients receiving an EGFR-targeted agent as part of their therapy.^39^

Prior to randomization, patients in the study were stratified according to first-line cancer therapy, type of EGFR-targeted agent prescribed or anticipated (TKI versus mAb), and the use of any concurrent medication that might be associated with increased sensitivity to sun exposure.^40^ Overall, 110 rash-free patients (39 lung, 45 CRC and 26 other malignancy) were randomized to receive an application of SPF60 sunscreen twice daily for 4 weeks versus placebo. Patients were monitored for rash and QoL using the skin-specific questionnaire Skindex-16 49 during the 4 weeks of topical application and for the 4 weeks following cessation of the treatment intervention.^40^ The primary objective of the study was to compare the incidence of rash in sunscreen- and placebo-treated patients. During the 4 weeks of intervention 78% and 80% of patients developed physician-reported skin rash in the sunscreen and placebo arms, respectively. Furthermore, no significant difference in the incidence of rash was reported for the subsequent 4 weeks. However, the incidence of higher-grade skin rash (grade 2 or >50% of body surface area) was lower, 33% versus 52% (p = 0.06), in the sun-screen arm of the study at 4 weeks.^40^

Although in this study, prophylactic treatment with sunscreen failed to reduce the overall incidence of skin rash, the fact that the incidence of higher-grade skin rash was markedly reduced in the sunscreen arm of the study led the study investigators to conclude that the prophylactic use of sunscreen should be continued in patients receiving EGFR-targeted therapy.

#### Tazarotene

A parallel study of topical tazarotene, a retinoic acid receptor specific retinoid, in the same patients as those investigated in the randomized minocyline trial, showed tazarotene to be ineffective with considerable patient attrition in the tazarotene portion of the study.^43^

#### Pimecrolimus

The authors of the randomized minocycline trial also conducted a randomized trial of the topical application of the immunomodulator pimecrolimus to one side of the face for patients with mCRC receiving cetuximab therapy^45^, and although the treated sides of the face had a greater decrease in lesion counts, this did not translate into a clinically meaningful benefit for patients.

#### Vitamin K

Vitamin K1 (phylloquinone) is naturally occurring. Vitamin K has been used for the treatment of blood vessel disorders of the skin, cosmetic skin treatment and skin treatment following laser treatment. It is thought to prevent disruption of the balance between proliferation and differentiation, thinning of the epidermis, immune reaction and inflammatory reactions leading to folliculitis. Topical vitamin K3 (Menadione) prevents erlotinib- and cetuximab-induced EGFR inhibition in the skin.^55^–^57^

To date, there have been four observational studies investigating the topical application of 0.1% vitamin K1 cream, three reactive 58–60 and one prophylactic.^61^ In all three reactive studies good control of EGFR-targeted therapy-induced rash was observed. In one study, in 79 mCRC patients premedicated with an H1 antagonist and corticos-teroids, receiving weekly cetuximab plus chemotherapy^59^, the topical application of a cream containing urea and 0.1% vitamin K1 twice daily on the appearance of acne-like rash (NCI-CTCAE v. 3.0), reduced the severity of the rash. The median time to improvement was 1.2 weeks and the median time to downstaging of the rash by ≥1 grade was 2.3 weeks.^59^ In another study, patients receiving cetuximab/panitumumab for the treatment of mCRC were treated with vitamin K1 cream at the first onset of grade ≥2 skin rash. The median duration of vitamin K1 cream treatment was 24 weeks (range, 6–28). Thirteen patients (39.4%) also received oral tetracycline therapy. A decrease in skin rash to grade 0–1 was observed in 12 (36.4%) patients, and 13 (39.4%) patients showed unchanged grade 2 skin toxicity. Overall, good skin rash symptom control was obtained in 69.2% of patients.^60^

In the prophylactic study^61^, the use of vitamin K1 cream (0.1% bid) applied to the face and chest was very effective at reducing the severity of cetuximab-induced skin toxicity (NCI-CTCAE 3.0) in 48 patients with mCRC receiving cetuximab in combination with chemotherapy, first-line. Application of vitamin K1 cream delayed the development of acne-like rash which peaked in the third week, and reduced the need for topical and systemic antibiotic treatment. All skin toxicities were grade 1/2 and no cetuximab dose reductions or treatment delays were required.

Thus, overall, topical vitamin K1 treatment has demonstrated significant clinical efficacy in the absence of any toxicity, with the limited evidence favoring a prophylactic treatment approach. Currently, there are no randomized trial data for the use of vitamin K therapy in the management of EGFR-targeted therapy-induced skin toxicities, although three randomized trials are ongoing (Table 4), one of which (EVITA), a double-blind, controlled, phase II study is evaluating the efficacy and safety of the prophylactic use of doxycycline +/− vitamin K cream in mCRC patients treated with chemotherapy plus cetuximab, first-line.

## Discussion

EGFR-agent-induced skin toxicities can be effectively treated at all stages and grade and are generally considered to be completely reversible except for telangiectasias. Their management is important and needs to be considered at as early a stage as possible as a prerequisite to maintaining patient QoL while continuing EGFR-targeted therapy. Evidence from two of the studies described above, with different treatment approaches, namely the STEPP study^16^ for interventions containing oral antibiotics and the prophylactic study of vitamin K1 cream^61^, suggest that prophylactic approaches should be the strategy of choice, but the agents of choice remain to be fully established as well as their schedule of administration. Certainly the data for doxycycline and vitamin K cream provide considerable cause for optimism in standardizing the management particularly of the early skin toxicities associated with therapy involving EGFR-targeted agents. The most recent guidelines by MASCC for the prevention of EGFR-targeted agent induced skin toxicities, recommend the preventive/prophylactic management of skin rash whenever possible, based on the observations from the randomized sunscreen trial^40^, and three of the randomized antibiotic trials.^16^,^41^,^43^ The recommended preventive therapy for the development of rash from the MASCC group would be the topical application of a steroid cream with moisturizer and sunscreen twice daily and systemic minocycline or doxycycline.^62^ Other studies that might be considered with a view to establishing and/or further defining the role of such treatment approaches in the management of EGFR-targeted agent-induced rash might include prophylactic doxycycline at different doses and randomized studies of:

Prophylactic doxycycline versus prophylactic vitamin K therapy

Prophylactic doxycycline plus prophylactic vitamin K therapy versus prophylactic vitamin K therapy versus prophylactic doxycycline therapy.

## Figures and Tables

**TABLE 1:** Summary of the different forms of cutaneous toxicities induced by epidermal growth factor receptor (EGFR)-targeted agents^8^,^12^,^14^

**Site**	**Description of cutaneous toxicity**
Skin	Acneiform skin rash
	Erythema
	Eczema
	Photosensitivity
	Fissures, rhagades
	Xerotic skin and pruritus
	Hyperpigmentation and teleangiectasia
Nails	Paronychia
	Fissures
Hair	Trichomegaly (growth of eyelashes)
	Hypertrichosis
	Alopecia
Eyes	Conjunctivitis
	Blepharitis
	Sicca syndrome, increased lacrimation

**TABLE 2:** Incidences of skin toxicity with EGFR-targeted agents in different licensed indications^8^,^63^–^67^

**Drug**	**Indication**	**Skin toxicities, %**	
		**All grades**	**Grade 3/4**
**EGFR-specific monoclonal antibodies**			
Cetuximab	**Metastatic *KRAS* wild-type CRC**		
	Monotherapy (after failure of 5-fluorouracil, oxaliplatin, irinotecan or intolerance of irinotecan)	90^a^	8^a^
	Plus irinotecan chemotherapy	88^a^	14^a^
	Combination with irinotecan-containing chemotherapy	NR	16.4
	Combination with oxaliplatin-containing chemotherapy	NR	11
	**SCCHN**		
	Combination with radiotherapy	87^a^	17^a^
	Combination with chemotherapy	NR	9
Panitumumab	**Metastatic *KRAS* wild-type CRC**		
	Monotherapy (after failure of fluoropyrimidine-, oxaliplatin- and irinotecan regimen)	90	16
	Combination with oxaliplatin-containing chemotherapy	22^a^	1^a^
	Combination with irinotecan-containing chemotherapy	96	36
		NR	37
**EGFR small molecule tyrosine kinase inhibitors**			
Erlotinib	Metastatic pancreatic cancer first-line in combination with gemcitabine	69^a^	5^a^
Erlotinib	Metastatic NSCLC – monotherapy (after failure of at least one prior chemotherapy regimen)	65	16
Gefitinib	Metastatic NSCLC – monotherapy (for continued treatment after failure of prior chemotherapy regimen)	47^a^	2^a^

a Rash only

EGFR = epidermal growth factor receptor; mCRC = metastatic colorectal cancer; NR = not reported; NSCLC = non-small cell lung cancer; SCCHN = squamous cell carcinoma of the head and neck

**TABLE 3:** Oral antibiotics in the treatment of skin rash

**Reference**	**Patients (n)**	**Patient characteristics**	**Antibiotic**	**Duration of skin treatment**	**Endpoint**	**Assessment tools**	**Skin toxicity Results** Placebo vs intervention	**Skin-related quality of life**
NCCTG N03CB-Jatoi et al., 2008^39^	61	Lung/gastrointestinal/other patients treated with gefitinib, cetuximab, erlotinib/other investigational agent	Tetracycline	500mg bid 4 weeks	Incidence of Grade >2 skin rash, QoL	NCI-CTCAE version 3.0, Skindex 16	76% vs 70% developed a rash. Grade 2 55% *vs* 17% at week 4	Less skin irritation, burning or stinging (Skindex-16) in tetracycline arm
Supplementary NCCTG N03CB-Jatoi et al., 2011^40^	65	As above	Tetracycline	500mg bid 4 weeks	Incidence of Grade >2 skin rash, QoL	NCI-CTCAE version 3.0, Skindex 16 and LASA.	Grade 2 identical	Identical (Skindex-16)
Scope et al., 2007^41^	48	mCRC patients treated with cetuximab	Minocycline	100mg/d 8 weeks	Total facial lesion counts;	Photography and patient-assessed rash severity and other cutaneous changes	Lower facial lesion count during weeks 1–4 in minocyline arm (p=0.005), Less severe facial rash (42% *vs 20%*	Less severe itching in minocycline arm, 50% *vs* 20% (p=0.05)
Lacouture et al., 2010^15^	95	Previously treated mCRC patients treated with panitumumab-containing therapy;	Doxycycline	100mg bid 6 weeks + skin moisturizer and sunscreen	Prophylactic vs reactive. incidence of protocol specified Grade >2 skin rash, QoL	NCI-CTCAE version 3.0, DLQI	Grade 2 reduced from 62% in the reactive group to 29% in the prophylactic group	Better (DLQI) in prophylactic group; change from baseline score less for prophylactic than reactive group
Deplanque et al., 2010^42^	147	Non-small cell lung cancer patients treated with erlotinib; all	Doxycycline	100 mg/d 4 months	Incidence of erlotinib folliculitis	NCI-CTCAE version 3.0	Incidence 82% vs 68%, Grade ≥2 reduced from 82% to 39%, also significant decrease in other cutaneous AEs.	na

AEs = adverse events; DLQI = Dermatological Life Quality Index; na = not available; NCI-CTCAE = National Cancer Institute Common Terminology Criteria for Adverse Events; mCRC = metastatic colorectal cancer

**TABLE 4:** Ongoing trials of Vitamin K in the management of skin toxicities induced by EGFR-targeted agents

**Trial**	**Planned patients (n)**	**Patient characteristics**	**Design**	**Primary endpoint**	**Secondary endpoint(s)**
EVITA NCT01345526	124	mCRC FOLFIRI + cetuximab	Vitamin K1 cream + Oral doxycycline versus Placebo + Oral doxycycline	Occurrence of acne-like skin rash grade >2 National Cancer Institute Common Terminology Criteria for Adverse Events version 4.0	Response Quality of life WoMo score 25
NCT0065678624	24	Patients with EGFR-targeted agent-induced rash	Menadione - vitamin K3 lotion Treatment-emergent versus Prophylactic	Safety and tolerability of vitamin K3 topical lotion	-
NCT01094444	36	Chemotherapy plus cetuximab in mCRC or squamous cell carcinoma of the head and neck patients	Prophylactic versus Reactive application of vitamin K3 lotion	Reduction of cutaneous side effects	Investigation of possible side-effects of lotion

EGFR = epidermal growth factor receptor; FOLFIRI = irinotecan, 5-fluorouracil, leucovorin; mCRC = metastatic colorectal cancer
